# Knowledge and Attitude towards Vaccination among Healthcare Workers: A Multicenter Cross-Sectional Study in a Southern Italian Region

**DOI:** 10.3390/vaccines8020248

**Published:** 2020-05-24

**Authors:** Giuseppe Di Martino, Pamela Di Giovanni, Arturo Di Girolamo, Piera Scampoli, Fabrizio Cedrone, Michela D’Addezio, Francesca Meo, Ferdinando Romano, Maria Bernadette Di Sciascio, Tommaso Staniscia

**Affiliations:** 1 Department of Medicine and Aging Sciences, “G. d’Annunzio” University Chieti-Pescara, Via dei Vestini 31, 66100 Chieti, Italy; tommaso.staniscia@unich.it; 2 Department of Pharmacy, “G. d’Annunzio” University Chieti-Pescara, Via dei Vestini 31, 66100 Chieti, Italy; pamela.digiovanni@unich.it; 3 Unit of Quality and Risk Management, ASL Lanciano-Vasto-Chieti, Via dei Vestini 31, 66100 Chieti, Italy; arturodigirolamo@gmail.com (A.D.G.); maria.disciascio@asl2abruzzo.it (M.B.D.S.); 4 School of Hygiene and Preventive Medicine, “G. d’Annunzio” University Chieti-Pescara, Via dei Vestini 31, 66100 Chieti, Italy; piera.scampoli@gmail.com (P.S.); cedronefab@gmail.com (F.C.); michela.daddezio@gmail.com (M.D.); francesca.meo10@gmail.com (F.M.); 5 Department of Public Health and Infectious Diseases, “La Sapienza” University of Rome, P.zza Aldo Moro 5, 00100 Rome, Italy; ferdinando.romano@uniroma1.it

**Keywords:** vaccination, vaccine hesitancy, healthcare workers, Italy, vaccine, attitudes

## Abstract

*Background*: In Italy, the loss of confidence towards vaccination resulted in low vaccine coverage, also among healthcare workers (HCWs). Indeed, low vaccination coverage among HCWs can lead to dangerous outbreaks of disease, reduce productivity, and increase absenteeism. The aim of this study was to investigate the vaccine coverage and attitudes toward vaccination among HCWs. *Methods*: A multicenter cross-sectional study was conducted among HCWs referred to all hospitals of the Local Health Authority 02 of Abruzzo Region, Italy. The survey was based on the questionnaire proposed by the H-ProImmune Project. Results: A total of 347 HCWs were enrolled in the study. Of these, 57.3% reported missing diphtheritis-tetanus-pertussis (DTP) vaccination, 50.1% reported missing measles-mumps-rubella (MMR) vaccination, and 62.5% reported missing flu vaccination. Regarding attitudes, other healthcare professionals reported to believe more in natural immunization compared to vaccination (26.5%; *p* < 0.001), and they were worried about long-term effects of vaccination (10.2%; *p* = 0.044). *Conclusions*: This survey showed all vaccination coverage considered resulted below the 95% threshold. Training on vaccination and mandatory measures may be needed in order to achieve better coverage.

## 1. Introduction

Vaccine-preventable diseases are a significant source of both morbidity and mortality in the general population. Vaccinations are universally recognized as one of the most effective preventive measures in public health [1]. Immunization programs can reduce the burden due to preventable infectious diseases and can decrease the related morbidity, mortality, and healthcare costs [2,3,4]. Achieving a 95% vaccination coverage rate is likely to avoid disease incidence and to protect also the unimmunized population segment [5]. The Global Vaccine Action Plan (2011–2020) (GVAP) is a framework adopted by all the World Health Organization (WHO) Member States at the Sixty-fifth World Health Assembly in 2012, aiming at a world free of vaccine-preventable diseases [6]. The European Vaccine Action Plan (2015–2020) (EVAP), developed by the 53 member states of the region together with the WHO Regional Office for Europe, immunization partners, and stakeholders, emphasizes the importance of implementing effective immunization policies throughout Europe [7]. In Italy, with the purpose of conforming to the regional strategies, the Ministry of Health has enacted the National Immunization Prevention Plan (PNPV). The PNPV is a guideline for the immunization policies developed to achieve all the vaccine coverages goals [8]. Over recent years, Europe has been facing the challenge of growing vaccine hesitancy: several countries and communities have been dealing with groups refusing the recommended vaccinations available for themselves and/or their children [9,10,11]. Vaccines are losing public confidence; thus, several international organizations (WHO, EU, ECDC) warn against the growing phenomenon of vaccine hesitancy and its impact on decreasing vaccine coverage trends [12,13,14]. This issue has created a need for national immunization programs that could find approaches and strategies to address vaccine hesitancy. In Italy, national low immunization coverage led to the introduction of compulsory vaccination in July 2017 for ten infectious diseases. Compulsory vaccination was introduced to protect public health and reach the target coverage of the PNPV [15,16]. Vaccine hesitancy is a phenomenon not only spreading among citizens but also among healthcare workers (HCWs). This has to be interpreted as a serious issue, since HCWs may spread infections to patients, colleagues, and relatives. Indeed, low vaccine coverages (VC) among HCWs can lead to dangerous disease outbreaks, reducing productivity and increasing absenteeism [17,18]. The World Health Organization (WHO) estimates that approximately 59 million HCWs worldwide are potentially exposed to hazardous biological agents daily [19]. The PNPV strongly recommends vaccination for HCWs [19]. Despite the new law about compulsory vaccination, in Italy there is no obligatory vaccination for HCWs. Although there is a lack of complete and certain data, vaccination coverage among HCWs is estimated to be very low [18].

The aim of this study was to evaluate the vaccine coverage and the attitudes towards vaccination among HCWs working in all the hospitals referred to the Local Health Authority (LHA) of Lanciano-Vasto-Chieti (LVC), Abruzzo Region, Italy.

## 2. Materials and Methods

A multicenter cross-sectional study was conducted in all the six hospitals and in all the ambulatory care structures referred to the LHA of LVC, Abruzzo Region, Italy. The online survey was based on the Italian version of the questionnaire proposed by the H-ProImmune Project [20]. The questionnaire was composed of three main sections: demographic characteristics, vaccine coverage, and attitudes and beliefs towards vaccination. The demographic section was composed of eight items exploring the demographic characteristics of the participants, their instruction level, and general information about their work. Nine items investigating the vaccination coverage of diphtheritis-tetanus-pertussis (DTP), Hepatitis B (HB), measles-mumps-rubella (MMR), and flu composed the vaccine coverage section. Twelve items about attitudes and beliefs were included in the section. Specifically, the answer of every item was based on a 5-point Likert scale. All the HCWs referred to the LHA were invited to participate in the survey via email. The study was conducted over the period between August and November 2019. Prior to the start, the study project was presented to all units involved by the Quality and Risk Management Unit referred to the LHA of LVC in July 2019. After the meeting, the link to the online survey was sent to all HCWs via email. In September, a reminder invitation was sent. All the questionnaires were anonymous, and participants provided informed consent to participate. The study was conducted according to the Declaration of Helsinki Ethical Principles for Medical Research Involving Human Subjects and respected the 2016/679 General Protection Data Regulation (GDPR) on privacy.

### Statistical Analysis and Sample Size Estimation

Considering the total of HCWs referred to the LHA of LVC (about 2500 HCWs), with a power level of 0.8 and an alpha error of 5%, a sample of 334 HCWs was required. Continuous variables were summarized as mean and standard deviation (SD) or median and interquartile range (IQR) according to their distribution. Categorical variables were summarized as frequencies and percentages. Kruskal–Wallis’ test was performed to compare continuous variables. Chi-Square tests or Fisher’s exact tests were performed to compare categorical variables as appropriate. Logistic regression analysis was performed to evaluate the association of type of occupation with vaccine coverage and attitudes towards vaccination, dichotomizing all the items included in the third section of the survey. Particularly, the answers reporting a score of 4 or 5 points in the Likert scale were considered as in “agreement” with the item, whereas scores between 1 and 3 were considered in “disagreement”. Association was expressed as odds ratios (OR) at a 95% confidence interval (95% CI). The false discovery rate correction (FDR) was used to control the family-wise type I error rate and an FDR adjusted *p*-value less than 0.05 was determined to be statistically significant. The analysis was performed with IBM SPSS for Statistics v.20.

## 3. Results

A total of 347 HCWs were enrolled in the study, 265 of which were females (76.4%), with a mean age of 50.2 ± 3.1 years. Most subjects involved were nurses (150, 43.2%) and working at the Hospital n. 1 (175, 50.4%), as showed in Table 1. 

The vaccination coverage rates are shown in Table 2. Specifically, 57.3% of the HCWs reported having received no vaccination against DTP over the last 10 years. The main reason for missing vaccination was the lack of knowledge about the necessity of a ten-year booster dose (56, 28.4%). A total of 20.3% of the HCWs considered DTP vaccination unnecessary. HB coverage reached 91.4% among the interviewed HCWs (*n* = 317). In total, 174 HCWs (50.1%) reported to be unvaccinated for measles-mumps-rubella (MMR), mainly because of previous natural immunization (125, 72.3%). Flu showed low coverage, with 217 HCWs (62.5%) that missed the vaccination. Several HCWs considered flu vaccination useless (124, 57.1%) or refused vaccination for personal reasons (38, 17.5%). Differences in vaccination coverage by age and gender were also found, as shown in the Supplementary Material (Tables S1 and S2). In particular, male HCWs were more likely to accept flu vaccination than female HCWs. In addition, all vaccination coverages showed a decreasing trend by age. 

Despite non-ideal vaccination coverages, most of interviewed HCWs suggested vaccination to their patients (231, 66.6%), even though 74 HCWs (21.3%) did not consider it as their competence (mainly other HCWs, 51; 68.9%). There were no differences in coverage by healthcare facility. On the contrary, Table 3 shows the differences in coverage resulting among different healthcare professionals. Particularly, midwives showed higher coverage for MMR (*p* = 0.002), and physicians showed higher coverage for flu (*p* < 0.001).

Table 4 outlines the attitudes about vaccination. The majority of HCWs considered vaccines important to prevent serious illnesses, with a lower agreement among nurses (*p* = 0.018). Other HCWs considered natural immunity more efficient compared with vaccine immunization (*p* = 0.001). Nurses and midwives reported feeling afraid of vaccination’s adverse effects (respectively 24.0% and 34.6%; *p* < 0.001). Moreover, nurses reported fear about becoming ill due to any disease (17.3%; *p* = 0.024), whereas midwives reported fear about becoming ill after vaccination (23.1%, *p* = 0.003). Mainly, physicians and other HCWs considered vaccinations a duty for all HCWs (90.2% and 89.8%; *p* < 0.001).

Table 5 shows the results of the logistic regression analyses. In comparison with physicians (considered as the reference in all the models), midwives were more likely to get vaccinated against MMR (OR: 2.8, 95%; CI: 1.1–7.6; *p* = 0.047). All other HCWs were less associated with flu vaccination than physicians. 

## 4. Discussion

This multicenter study aimed to investigate vaccine coverages and attitudes towards vaccination in a sample of HCWs from different settings. HCWs are particularly exposed to vaccine-preventable diseases and can play a role in hospital transmission, which makes them an important target group for vaccination. The results of this study confirmed low vaccine coverages among HCWs, as reported in previous studies [18,21,22,23,24].

According to our results, all HCWs do not reach 95% vaccine coverage for all the four vaccinations chosen for this survey. Beside vaccination coverage, the reasons for missing vaccination were investigated. HCWs frequently reported missing their booster dose of DTP because of lack of knowledge (28.4%), because the booster was considered useless (20.3%), due to forgetfulness (16.8%), or due to natural immunity (5.5%). These reasons show an important gap in disease knowledge, highlighting the need for adequate training programs for HCWs, together with mandatory measures. The same considerations should be made about HB vaccination: About 10% of HCWs did not receive vaccination, and the greatest part of them considered it unnecessary (59.3%). The results about MMR vaccination were predictable, as it was the vaccine most frequently involved in vaccine hesitancy [25,26]. This is in line with the recent literature, showing an increase in the occurrence of nosocomial measles episodes in Italy, as reported by Porretta et al. [27] and Amendola et al. [28]. A great part of HCWs who missed vaccination reported to be immunized (72.3%). This point should be monitored by hospital directors and by occupational medicine physicians as the MMR vaccine was only recently introduced (in 1976, a single antigen live attenuated measles vaccine was introduced by the Italian Ministry of Health, and only in 1999, a combined MMR vaccine was included in the national immunization schedule [29]), and it is possible that many HCWs were naturally immunized. Despite this, it is not probable that all the HCWs who reported to be immunized had experienced all the three diseases covered by the MMR vaccine. As reported by previous studies [18,21,22,23,24], low coverages among all HCW categories were also reported for flu vaccination. In line with previous studies, the main reason for missing flu vaccination is the supposed uselessness of this vaccine, in line with previous studies [30,31,32]. This point highlights the need for focused training in order to iMMRove the knowledge about flu vaccination. Important differences in vaccination coverages were found by age and gender. In particular, older HCWs were less likely to accept vaccination, in contrast with findings of previous studies [33]. The main reasons for these findings can be found in less knowledge and poor confidence in vaccination. Moreover, outside Europe, low coverage among HCWs is still a public health concern, as confirmed by Khan et al. [34]. Multivariate analyses showed that all the HCWs were less likely to be vaccinated for flu compared to physicians, confirming the assumption that poor knowledge about flu has an impact on patients. Midwives were significantly associated with MMR vaccination compared to physicians. These results are probably due to their deep knowledge about the impact of these diseases on pregnant women and newborns. A broad sentiment of vaccine hesitancy has grown throughout Europe [12,35] over the last decade. This sentiment is present also among HCWs [36,37], highlighting the lack of confidence in vaccine safety and usefulness. Fear of vaccine side effects, fear of becoming sick after vaccination, and the conviction to be immune against all the infectious diseases are attitudes that cannot be justified among HCWs. These outcomes have already been highlighted in previous studies [37,38,39], showing growing vaccine hesitancy also among HCWs. In order to fight vaccine hesitancy among HCWs, it is essential to promote clear and effective communication involving vaccinations and to adopt innovative strategies such as promoting vaccination via social networks [37,40] or developing focused training for HCWs [18]. In order to increase vaccination coverage among HCWs, in 2017, a multidisciplinary group of Italian experts developed a position paper entitled *The Pisa Charter on HCWs Vaccination*, signed by seven Italian scientific societies. In this document, the need to promote vaccination practice and the importance of HCWs’ vaccination were outlined [41]. Despite this, vaccination coverage among HCWs remains low, representing a challenging issue for public health. In order to reach the vaccination coverage target, some Italian regions have introduced mandatory vaccination for HCWs. In addition, the fact that the COVID-19 epidemic occurred during the first part of the year 2020 can help in iMMRoving vaccination among HCWs. In particular, flu vaccination coverage can be enhanced in order to facilitate the differential diagnosis in cases of new COVID-19 outbreak during the next winter season.

### Strengths and Limitation

The strength of this study is the adequate number of HCWs interviewed, working in different healthcare settings with different working statuses. Using a validated tool, this study focused both on vaccination coverage and on attitudes and beliefs towards vaccines. The main limitation was the unequal distribution of HCWs by working role and by healthcare setting. In addition, data were self-reported, and all the HCWs enrolled were referred to the same LHA.

## 5. Conclusions

Mandatory measures may be needed in order to achieve better vaccination coverage. A vaccinated HCW protects himself, patients, and relatives. In particular, vaccinated HCWs could avoid increasing the public health expenditure and worsening the health condition of oncological and immunocoMMRomised patients [39]. In addition, increasing vaccine confidence among HCWs may allow a more effective fight against vaccine hesitancy among the general population. 

## Figures and Tables

**Table 1:** Healthcare worker (HCW) characteristics.

*N* = 347	Physicians (*n* = 122)	Nurses (*n* = 150)	Midwives (*n* = 26)	Others (*n* = 49)	*p*-Value ^a^
**Gender**					**0.002**
Female	82 (67.2)	120 (80.0)	26 (100)	37 (75.5)	
Male	40 (32.8)	30 (20.0)	-	12 (24.5)	
**Age**					**<0.001**
<30	13 (10.7)	3 (2.0)	3 (11.5)	3 (6.1)	
30–40	27 (22.1)	18 (12.0)	14 (53.8)	7 (14.3)	
41–50	29 (23.8)	53 (35.3)	6 (23.1)	14 (28.6)	
51–60	27 (22.1)	69 (46.0)	3 (11.5)	16 (32.7)	
>60	26 (21.3)	7 (4.7)	-	9 (18.4)	
**Level of instruction**					**<0.001**
Master’s degree/PhD	122 (100)	34	2	19 (38.8)	
Bachelor’s degree	-	60	22	12 (24.5)	
Others	-	56	-	18 (36.7)	
**Workplace**					**<0.001**
Hospital 1	73 (59.8)	62 (41.3)	23 (88.5)	17 (34.7)	
Hospital 2	12 (9.8)	45 (30.0)	-	8 (16.3)	
Ambulatory healthcare facilities	13 (10.7)	18 (12.0)	-	15 (30.6)	
Hospital 3	7 (5.7)	14 (9.3)	2 (7.7)	2 (4.1)	
Others	17 (13.9)	11 (7.3)	1 (3.8)	7 (14.3)	
**Years of work median (IQR)**	**14 (4–28)**	**25 (19–32)**	**13 (9–19)**	**19 (10–28)**	**<0.001 ^b^**

^a^ Chi-square test; ^b^ Kruskal–Wallis test; bolded *p*-values were significant after false discovery rate correction (FDR) correction.

**Table 2:** Vaccination coverages and reasons for missing vaccination.

Questions	N (%)
**During the last 10 years, did you perform** **DTP booster dose?**	
Yes	122 (32.3)
No	199 (57.3)
Don’t remember	36 (10.4)
**Reasons for missing vaccination**	
No knowledge of booster dose need	56 (28.4)
Useless/Not necessary	40 (20.3)
Forgotten	33 (16.8)
Vaccination made during childhood	23 (11.7)
Natural immunization	19 (9.6)
Others	26 (13.2)
**Did you ever perform HB vaccination?**	
Yes	317 (91.4)
No	27 (7.8)
Don’t remember	3 (0.9)
**Reasons of for missing vaccination**	
Useless/Not necessary	16 (59.3)
Forgotten	5 (18.5)
Others	6 (22.2)
**Did you ever perform** **MMR vaccination?**	
Yes	153 (44.1)
No	174 (50.1)
Don’t remember	20 (5.8)
**Reasons for missing vaccination**	
Useless/Not necessary	18 (10.4)
Forgotten	11 (6.3)
Natural immunization	125 (72.3)
Others	19 (11.0)
**Did you perform seasonal flu vaccination?**	
Yes	122 (35.2)
No	217 (62.5)
Don’t remember	8 (2.3)
**Reasons for missing vaccination**	
Useless/Not necessary	124 (57.1)
Forgotten	5 (2.3)
Personal choose	38 (17.5)
Others	50 (23.1)

**Table 3:** Vaccination coverages by working status.

Vaccines	Physician N (%)	Nurse N (%)	Midwives N (%)	Others N (%)	*p*-Value ^a^
DTP	44 (36.1)	46 (30.7)	10 (38.5)	12 (24.5)	0.413
HB	109 (89.3)	144 (96.0)	26 (100.0)	38 (77.6)	**0.004**
MMR	55 (45.1)	65 (43.3)	20 (76.9)	13 (26.5)	**0.002**
Flu	71 (58.2)	31 (20.7)	3 (11.5)	17 (34.7)	**<0.001**

Abbreviations: DTP = diphtheritis-tetanus-pertussis; HB = Hepatitis B; MMR = measles-mumps-rubella; ^a^ chi-square test; Bolded *p*-values were significant after *FDR* correction.

**Table 4:** Attitudes and beliefs towards vaccination.

Questions	Physicians N (%)	Nurses N (%)	Midwives N (%)	Others N (%)	*p*-Value ^a^
I believe vaccines are important for reducing or eliminating serious diseases	120 (98.4)	137 (91.3)	26 (100.0)	48 (98.0)	0.018
I believe vaccines are useful in certain situations, for example in developing countries	114 (93.4)	132 (88.0)	26 (100.00)	46 (93.9)	0.121
I believe more in natural immunity acquired through disease than in vaccines	6 (4.9)	24 (16.0)	3 (11.5)	13 (26.5)	**0.001**
I think vaccinations do more harm than good	1 (0.8)	9 (6.0)	-	3 (6.1)	0.079
I’m afraid of the side effects of vaccinations	7 (5.7)	36 (24.0)	9 (34.6)	7 (14.3)	**<0.001**
My religious beliefs are against vaccinations	1 (0.8)	4 (2.7)	-	1 (2.0)	0.603
I don’t think I’m at risk of contracting any infectious disease	7 (5.7)	26 (17.3)	2 (7.7)	5 (10.2)	*0.024*
I’m afraid of getting sick after getting vaccinated	3 (2.5)	17 (11.3)	6 (23.1)	5 (10.2)	**0.003**
I believe vaccines are not effective	3 (2.5)	9 (6.0)	-	4 (8.2)	0.203
I am wary of the long-term health effects of vaccinations	2 (1.6)	10 (6.7)	-	5 (10.2)	*0.044*
I believe that vaccinations among HCWs are a prerequisite for working in the healthcare sector	108 (88.5)	111 (74.0)	22 (84.6)	42 (85.7)	**0.016**
I think vaccinations are a duty of HCWs because they should be a model for patients	110 (90.2)	98 (65.3)	20 (76.9)	44 (89.8)	**<0.001**

^a^ Chi-square test; bolded *p*-value were significant after FDR correction.

**Table 5:** Multivariable logistic regression models to assess the likelihood of vaccination by working status.

	OR *	IC95%	*p*-Value *
**DTP**			
Physicians (Reference)	1		
Nurses	1.0	0.6–2.2	0.624
Midwives	1.0	0.4–3.1	0.766
Others	0.6	0.3–1.4	0.252
**HB**			
Physicians (Reference)	1		
Nurses	1.1	0.8–16.5	0.102
Midwives	1.3	0.7–10.6	0.324
Others	0.6	0.4–1.9	0.676
**MMR**			
Physicians (Reference)	1		
Nurses	1.4	0.7–2.7	0.306
Midwives	**2.8**	**1.1–7.6**	0.047
Others	0.9	0.7–1.4	0.980
**Flu**			
Physicians (Reference)	1		
Nurses	**0.2**	**0.1–0.3**	**<0.001**
Midwives	**0.1**	**0.1–0.5**	**0.003**
Others	**0.4**	**0.2–0.8**	**0.012**

Abbreviations: DTP = diphtheritis-tetanus-pertussis; HB = Hepatitis B; MMR = measles-mumps-rubella; OR = odds ratio; 95% CI = 95% confidence interval. * All models were adjusted by age, gender, instruction level and years of work; bolded *p*-values were significant after FDR correction.

## Supplementary Materials

The following are available online at https://www.mdpi.com/2076-393X/8/2/248/s1, Table S1: Differences in vaccination coverage by gender, Table S2: Differences in vaccination coverage by age.

## References

[1] Andre F.E., Booy R., Bock H.L., Clemens J., Datta S.K., John T.J., Lee B.W., Lolekha S., Peltola H., Ruff T.A. (2008). Vaccination greatly reduces disease, disability, death and inequity worldwide. Bull. World Health Organ..

[2] Orensteina W.A., Ahmedb R. (2017). Simply put: Vaccination saves lives. Proc. Natl. Acad. Sci. USA.

[3] Roush S.W., Murphy T.V., Basket M.M., Iskander J.K., Moran J.S., Seward J.F., the Vaccine-Preventable Disease Table Working Group (2007). Historical comparisons of morbidity and mortality for vaccine-preventable diseases in the United States. J. Am. Med. Assoc..

[4] CDC (2015). Epidemiology and Prevention of Vaccine-Preventable Diseases.

[5] Johni T.J., Samuell R. (2000). Herd Immunity and Herd Effect: New Insights and Definitions. Eur. J. Epidemiol..

[6] WHO Global Vaccine Action Plan 2011–2020 [Internet]. http://www.who.int/immunization/global_vaccine_action_plan/GVAP_doc_2011_2020/en/).

[7] World Health Organization Regional Office for Europe (2014). European Vaccine Action Plan 2015–2020.

[8] Signorelli C., Guerra R., Siliquini R., Ricciardi W. (2017). Italy’s response to vaccine hesitancy: An innovative and cost effective National Immunization Plan based on scientific evidence. Vaccine.

[9] Nowak G., Gellin B., MacDonald N.E., Butler R. (2015). Addressing vaccine hesitancy: The potential value of commercial and social marketing principles and practices. Vaccine.

[10] Larson H.J., Jarrett C., Eckersberger E., Smith D.M.D., Paterson P. (2014). Understanding vaccine hesitancy around vaccines and vaccination from a global perspective: A systematic review of published literature, 2007. Vaccine.

[11] Dubé E., Vivion M., MacDonald N.E. (2014). Vaccine hesitancy, vaccine refusal and the anti-vaccine movement: Influence, impact and implications. Expert Rev. Vaccines.

[12] Gualano M.R., Bert F., Voglino G., Buttinelli E., D’Errico M.M., De Waure C., Di Giovanni P., Fantini M.P., Giuliani A.R., Marranzano M. (2018). Attitudes towards compulsory vaccination in Italy: Results from the NAVIDAD multicentre study. Vaccine.

[13] Hickler B., Guirguis S., Obregon R. (2015). Vaccine Special Issue on Vaccine Hesitancy. Vaccine.

[14] Odone A., Signorelli C. (2017). When vaccine hesitancy makes headlines. Vaccine.

[15] Di Martino G., Di Giovanni P., Staniscia T. (2017). The Italian Action Against Vaccine Hesitancy. SM Prev. Med. Public Health.

[16] Italian Official Gazette, I Legge n.119/Disposizioni Urgenti in Materia di Prevenzione Vaccinale. [Internet]. http://www.gazzettaufficiale.it/eli/id/2017/08/5/17G00132/sg.

[17] Gianino M.M., Politano G., Scarmozzino A., Charrier L., Testa M., Giacomelli S., Giacomelli S., Benso A., Zotti C.M. (2017). Estimation of sickness absenteeism among Italian healthcare workers during seasonal influenza epidemics. PLoS ONE.

[18] Genovese C., Picerno I.A.M., Trimarchi G., Cannavò G., Egitto G., Cosenza B., Merlina V., Icardi G., Panatto D., Amicizia D. (2019). Vaccination coverage in healthcare workers: A multicenter cross-sectional study in Italy. J. Prev. Med. Hyg..

[19] WHO Occupational Health. Health Workers. Health Worker Occupational Health [Internet]. http://www.who.int/occupational_health/topics/hcworkers/en.

[20] European Union HProImmune Project [Internet]. http://www.hproimmune.eu/.

[21] Barchitta M., Basile G., Lopalco P.L., Agodi A. (2019). Vaccine-preventable diseases and vaccination among Italian healthcare workers: A review of current literature. Future Microbiol..

[22] Alicino C., Iudici R., Barberis I., Paganino C., Cacciani R., Zacconi M., Battistini A., Bellina D., Di Bella A.M., Talamini A. (2015). Influenza vaccination among healthcare workers in Italy: The experience of a large tertiary acute-care teaching hospital. Hum. Vaccines Immunother..

[23] Fortunato F., Tafuri S., Cozza V., Martinelli D., Prato R. (2015). Low vaccination coverage among italian healthcare workers in 2013: Contributing to the voluntary vs. mandatory vaccination debate. Hum. Vaccines Immunother..

[24] Squeri R., Genovese C., Trimarchi G., Palamara M.A.R., La Fauci V. (2017). An evaluation of attitude toward vaccines among healthcare workers of a University Hospital in Southern Italy. Ann di Ig.

[25] Hotez P.J., Nuzhath T., Colwell B. (2020). Combating vaccine hesitancy and other 21st century social determinants in the global fight against measles. Curr. Opin. Virol..

[26] Gasparini R., Panatto D., Lai P.L., Amicizia D. (2015). The “urban myth” of the association between neurological disorders and vaccinations. J. Prev. Med. Hyg..

[27] Porretta A., Quattrone F., Aquino F., Pieve G., Bruni B., Gemignani G., Vatteroni M.L., Pistello M., Privitera G.P., Lopalco P.L. (2017). A nosocomial measles outbreak in Italy, February–April 2017. Eurosurveillance.

[28] Amendola A., Bianchi S., Frati E.R., Ciceri G., Faccini M., Senatore S., Colzani D., Lamberti A., Baggieri M., Cereda D. (2017). Ongoing large measles outbreak with nosocomial transmission in Milan, northern Italy, March–August 2017. Eurosurveillance.

[29] Pezzotti P., Bellino S., Prestinaci F., Iacchini S., Lucaroni F., Camoni L., Barbieri M.M., Ricciardi W., Stefanelli P., Rezza G. (2018). The impact of immunization programs on 10 vaccine preventable diseases in Italy: 1900. Vaccine.

[30] Boey L., Bral C., Roelants M., De Schryver A., Godderis L., Hoppenbrouwers K., Vandermeulen C. (2018). Attitudes, believes, determinants and organisational barriers behind the low seasonal influenza vaccination uptake in healthcare workers—A cross-sectional survey. Vaccine.

[31] Riphagen-Dalhuisen J., Gefenaite G., Hak E. (2012). Predictors of seasonal influenza vaccination among healthcare workers in hospitals: A descriptive meta-analysis. Occup. Environ. Med..

[32] Harrison N., Brand A., Forstner C., Tobudic S., Burgmann K., Burgmann H. (2016). Knowledge, risk perception and attitudes toward vaccination among Austrian health care workers: A cross-sectional study. Hum. Vaccines Immunother..

[33] Pichon M., Gaymard M., Zamolo H., Bazire C., Valette M., Sarkozy F., Lina B. (2019). Web-based analysis of adherence to influenza vaccination among French healthcare workers. J. Clin. Virol..

[34] Khan T., Khan A., Ali I., Wu D. (2016). Knowledge, attitude and awareness among healthcare professionals about influenza vaccination in Peshawar, Pakistan. Vaccine.

[35] Bert F., Olivero E., Rossello P., Gualano M.R., Castaldi S., Damiani G., D’Errico M.M., Di Giovanni P., Fantini M.P., Fabiani L. (2019). Knowledge and beliefs on vaccines among a sample of Italian pregnant women: Results from the NAVIDAD study. Eur. J. Public Health.

[36] Paterson P., Meurice F., Stanberry L.R., Glismann S., Rosenthal S.L., Larson H.J. (2016). Vaccine hesitancy and healthcare providers. Vaccine.

[37] Yaqub O., Castle-Clarke S., Sevdalis N., Chataway J. (2014). Attitudes to vaccination: A critical review. Soc. Sci. Med..

[38] Guthmann J.P., Fonteneau L., Ciotti C., Bouvet E., Pellissier G., Lévy-Bruhl D., Abiteboul D. (2012). Vaccination coverage of health care personnel working in health care facilities in France: Results of a national survey. Vaccine.

[39] La Torre G., Scalingi S., Garruto V., Siclari M., Chiarini M., Mannocci A. (2017). Knowledge, Attitude and Behaviours towards Recommended Vaccinations among Healthcare Workers. Healthcare.

[40] Stahl J.P., Cohen R., Denis F., Gaudelus J., Martinot A., Lery T., Lepetit H. (2016). The impact of the web and social networks on vaccination. New challenges and opportunities offered to fight against vaccine hesitancy. Med. Mal. Infect..

[41] The Pisas’ Paper of Vaccinations in Healthcare Professionals [Internet]. https://gimpios.it/r.php?v=2904&a=29262&l=334060&f=allegati/02904_2017_04/fulltext/155-157_CartaPisa.pdf.

