# Supplementary material for: Knowledge and Attitude towards Vaccination among Healthcare Workers: A Multicenter Cross-Sectional Study in a Southern Italian Region
Source: Vaccines (Basel). 2020 May 24;8(2):248. doi: 10.3390/vaccines8020248 (PMC7350011; doi:10.3390/vaccines8020248)
Supplement: Supplementary file 1 [file vaccines-08-00248-s001.pdf]

Table S1. Differences in vaccination coverage by gender

Gender	DTP	p-value	HB	p-value	MPR	p-value	Flu	p-value
**Female**	79 (29.8)	0.077	243 (91.7)	0.682	111(41.9)	0.137	76 (28.7)	<0.001
**Male**	33 (40.2)		74 (90.2)		42 (51.2)		46 (56.1)	

Table S2. Differences in vaccination coverage by age

Age class	DTP	p-value	HB	p-value	MPR	p-value	Flu	p-value
**<30**	14 (63.6)		22 (100)		20 (90.9)		9 (40.9)	
**30-40**	26 (39.4)		66 (100)		48 (72.7)		24 (36.4)	
**41-50**	41 (40.2)	<0.001	92 (90.2)	0.017	51 (50.0)	<0.001	26 (25.5)	0.001
**51-60**	23 (20.0)		101 (87.8)		27 (23.5)		37 (32.2)	
**>60**	8 (19.0)		36 (85.7)		7 (16.7)		26 (61.9)	
